# Nanoparticles for Creating a Strategy to Stimulate Liver Regeneration

**DOI:** 10.17691/stm2024.16.3.04

**Published:** 2024-06-28

**Authors:** S.A. Rodimova, D.S. Kozlov, D.P. Krylov, L.V. Mikhailova, V.A. Kozlova, A.I. Gavrina, A.M. Mozherov, V.V. Elagin, D.S. Kuznetsova

**Affiliations:** Junior Researcher, Research Laboratory of Regenerative Medicine; Research Laboratory of Molecular Biotechnologies, Institute of Experimental Oncology and Biomedical Technologies; The Institute of Experimental Medicine, 12 Akademika Pavlova St., Saint Petersburg, 197376, Russia; Laboratory Assistant, Research Laboratory of Molecular Biotechnologies, Institute of Experimental Oncology and Biomedical Technologies; The Institute of Experimental Medicine, 12 Akademika Pavlova St., Saint Petersburg, 197376, Russia; Student; National Research Lobachevsky State University of Nizhny Novgorod, 23 Prospekt Gagarina, Nizhny Novgorod, 603022, Russia; Laboratory Assistant, Research Laboratory of Molecular Biotechnologies, Institute of Experimental Oncology and Biomedical Technologies; The Institute of Experimental Medicine, 12 Akademika Pavlova St., Saint Petersburg, 197376, Russia Student; National Research Lobachevsky State University of Nizhny Novgorod, 23 Prospekt Gagarina, Nizhny Novgorod, 603022, Russia; Engineer, Department of Physics; ITMO University (Saint Petersburg National Research University of Information Technologies, Mechanics and Optics), 49 Kronverksky Pr., Saint Petersburg, 197101, Russia; Student; National Research Lobachevsky State University of Nizhny Novgorod, 23 Prospekt Gagarina, Nizhny Novgorod, 603022, Russia; Junior Researcher, Research Laboratory of Molecular Biotechnologies, Institute of Experimental Oncology and Biomedical Technologies; The Institute of Experimental Medicine, 12 Akademika Pavlova St., Saint Petersburg, 197376, Russia; Junior Researcher, Research Laboratory of Optical Spectroscopy and Microscopy, Institute of Experimental Oncology and Biomedical Technologies; The Institute of Experimental Medicine, 12 Akademika Pavlova St., Saint Petersburg, 197376, Russia; PhD, Researcher, Research Laboratory of Optical Spectroscopy and Microscopy; Research Laboratory of Molecular Biotechnologies, Institute of Experimental Oncology and Biomedical Technologies; The Institute of Experimental Medicine, 12 Akademika Pavlova St., Saint Petersburg, 197376, Russia; PhD, Head of the Research Laboratory of Molecular Biotechnologies, Institute of Experimental Oncology and Biomedical Technologies; The Institute of Experimental Medicine, 12 Akademika Pavlova St., Saint Petersburg, 197376, Russia

**Keywords:** nanoparticles, biodistribution, FLIM, liver slices, liver cell culture, liver regeneration

## Abstract

Presently, there is a need in the developing new approaches to stimulate liver regeneration, which would make its recovery more effective after resection. Application of nanoparticles, loaded with small bioactive molecules, with their targeted delivery into the liver is a promising approach.

**The aim of the investigation** is to study the interaction of nanoparticles with various types of hepatic cells on the models of liver slices and primary hepatic cell cultures using the methods of multiphoton microscopy with fluorescence lifetime imaging.

**Materials and Methods:**

Nanoparticles have been synthetized from polylactide (PLA), gold (Au), and silicon (SiO_2_), and characterized using scanning and transmission electron microscopy. These types of particles were labeled with a fluorescent Cy5 dye for their visualization. Liver slices and a primary hepatocyte culture were used as models for biological testing of nanoparticles. Biodistribution of the nanoparticles in the tissue and cells, their cytotoxicity, and the effect on the cell metabolism were assessed using optical bioimaging methods.

**Results:**

The silicon nanoparticles are accumulated mainly by macrophages, which generate reactive oxygen species in a large amount and impair the native metabolic state of hepatocytes. The gold nanoparticles accumulate in all types of the liver cells but possess a marked toxic effect, which is indicated by the appearance of necrotic and apoptotic cells and a sharp change in the hepatocyte metabolic state. The polylactide nanoparticles accumulate most effectively in the liver cells, preferably in hepatocytes, do not change their native metabolic state, making this type of nanoparticles most promising for creating the bioactive molecule delivery systems to stimulate liver regeneration.

## Introduction

Currently, a high risk of postoperative liver insufficiency remains a serious problem in patients with comorbid liver pathology after extensive resections. Over 50% of patients undergone liver resection and 25% of donors for liver transplantation have been known to have some degree of steatosis and fibrosis [1], which reduces significantly the liver regenerative potential. Therefore, to develop a new approach to stimulation of liver regeneration, which would improve the efficiency of its recovery after resection, is of great importance.

The stimulation of liver regeneration using various small bioactive molecules, which act both at the level of a molecule and the entire organ, seems to be a promising approach to solve this problem [2, 3]. However, despite the optimistic results achieved by the present time, the problem of biomolecule delivery with the controlled release, accumulation, and elimination remains unsolved. Multiple studies have shown that nanoparticles (NP) are capable of transporting efficiently different bioactive molecules, for example, microRNA, to the target organs [4, 5].

Today, three types of NPs are widely used in biomedical investigations: silicon (SiO_2_), polylactide (PLA), and gold (Au). NPs with a high surface area/ volume ratio provide a simple conjugation with the therapeutic molecules [6, 7]. Besides, by changing the NP size and shape, it is possible to control their properties such as the rate of releasing bioactive molecules, time of accumulation, and excretion from the cells. Of special importance is the fact that no complex surface modifications are required for NP delivery to the target cells, namely the hepatocytes, owing to the ability of almost all NP types to passively accumulate in the liver cells.

Generally, physical and chemical properties of these NPs are well studied but their biological effect remains in many respects unclear. Besides, it has not yet been established, which particular type of liver cells uptake a certain type of NPs. Hepatocytes, which are responsible for the majority of metabolic processes of the liver and entire organism, are the most preferable for accumulation of bioactive complexes, as well as stellate cells, which are capable of transition into myofibroblasts synthetizing and secreting proteins of the extracellular matrix in acute or chronic liver injury [8, 9]. Both types of cells are suitable targets for stimulation of liver regeneration and/or therapy of comorbid liver pathology.

A metabolic state of the cells is a sensitive marker of their condition. Presently, methods of multiphoton microscopy are actively used in biomedical investigations to explore the metabolic cell activity by fluorescence intensity of nicotinamide adenine dinucleotide (NADH). Besides, fluorescence lifetime imaging microscopy (FILM) allows data acquisition on the amount of time the molecule remains in the excited state and contributions of a free and bound forms of the NADH, which are involved in the main energetic metabolic pathways: glycolysis and oxidative phosphorylation [10].

Thus, this work is aimed at studying the interaction of PLA, SiO_2_, and Au nanoparticles with various types of liver cells on the models of liver slices and primary liver cell cultures using multiphoton microscopy with fluorescence lifetime detection.

## Materials and Methods

### Nanoparticle synthesis

The following materials were used for carrier synthesis: SiO_2_ NPs — solution of ammonium hydroxide (NH_4_OH, 27%), tetraethyl-orthosilicate (tetraethoxysilane, TEOS, 99%), ethanol (EtOH, 99.9%; Merck, USA); PLA NPs — polylactide (PLA, MW=60,000; Goodfellow, England), dichloromethane (0.08% of ethanol by mass; Ecos-1, Russia), acetone (99.5%; PanReac AppliChem, USA); Au NPs — borohydride (NaBH_4_), hydrogen tetrachloroaurate (III) (HAuCl_4_), cetyltrimethylammonium bromide (CTAB).

The SiO_2_ NPs were synthetized by the sol-gel process using the standard Stӧber method [11]. For PLA NPs production, we employed a modified method of a well-known nanoprecipitation technique [12]. The obtained NPs were analyzed using high-resolution scanning electron microscopy (SEM) (Merlin, Carl Zeiss, Germany) at accelerating voltage of 0.02–30.0 kV. Before measurements, 1.5 μl of each type of NPs, dispersed in water, was applied on the cover glass and left to dry for 30 min. The dry samples were visualized using SEM.

Precision measurements were performed by means of a high-resolution Zeiss Libra 200F transmission electron microscope (TEM) (Carl Zeiss, Germany) with 120– 200 kV accelerating voltage equipped with an autoemitter and OMEGA energy filter (OMEGA AIR, Slovenia).

### Fabrication of liver slices

Liver slices were obtained from the 0.5×0.5 cm fresh tissue samples using 7000smz-2 vibrating microtome (Campden Instruments Ltd., Great Britain) having the following technical characteristics: 80 Hz frequency, 2 mm oscillation amplitude, 0.4 mm/s blade speed, 400 μm pitch size (tissue explant thickness). The samples were preliminarily fixed on the microtome table. Immediately after fabrication, every slice was placed in the PBS buffer on ice. Further, freshly prepared liver slices were individually transferred to the well of the 12-well plate with 2 ml of the standard cultural medium for preincubation for 1 h. The standard cultural medium was composed of DMEM (PanEco, Russia) with addition of 10% FBS (Gibco, USA), which provides formation of corona from superficial biomolecules [13], 4 mM L-glutamine (PanEco, Russia), 0.1 μM dexamethasone (StemCells Technologies, USA), and antibiotic-antimycotic consisting of 100 units/ml of penicillin, 100 μg/ml of streptomycin, and 25 μg/ml of Fungizone (Gibco, USA).

After the incubation, the culture medium was changed. Further cultivation lasted for 3 h. The medium was again changed and incubation continued for all liver slices for 24 and 48 h from the beginning of cultivation under standard conditions of CO_2_-incubator.

### Preparation of primary liver cell cultures

A primary liver cell culture was obtained following the modified protocol described by Kegel et al. [14] using collagenase, type II (Sigma, USA), for removing intracellular substance and adding lysing buffer solution at ×10 concentration for removing erythrocytes from cell suspension. The two obtained separate fractions of hepatocytes and non-parenchymal cells were purified from cellular debris and placed into the wells of the 12-well plate (10^4^ hepatocytes per 2 ml of the standard cultural medium). Further cultivation was carried out under the standard conditions of the CO_2_-incubator.

### Assessment of nanoparticle biodistribution in the primary cell culture

All three types of NPs were labeled with a fluorescent Cy5 dye (excitation at 633 nm wavelength, emission at 670 nm wavelength) following the method described in the work [15]. All dyes were used in the form of activated NHS Ester for binding to BSA (bovine serum albumin). To assess the biodistribution of the Cy5-labeled NPs, cell nuclei in the liver cells (Hoechst; PanEco, Russia; excitation at 405 nm, emission at 457 nm) and lysosomes (LysoTracker Yellow HCK-123; Invitrogen, USA; excitation at 465 nm, emission at 535 nm) were stained according to the manufacturers’ instructions.

### Assessment of liver cell viability on the model of liver slices and primary cell culture

The viability of the obtained liver slices and primary cell culture was validated by staining with calcein and propidium iodide in compliance with the manufacturer’s protocol (Live/ Dead Cell Double Staining Kit; Thermo Fisher Scientific, USA), cell nuclei were stained with the Hoechst stain. Microscopic examination was performed using LSM 880 microscope (Carl Zeiss, Germany). Images were acquired with 1024×1024 pixel resolution and by two scan averaging. For calcein, the excitation was at 488 nm wavelength, the signal was recorded in the range of 500–570 nm; for propidium iodide, excitation was at 543 nm wavelength, the signal was recorded in the range of 600–700 nm; for Hoechst stain, the excitatation was at 405 nm, the signal was registered in the range of 457 nm. Ten fields of view were obtained for each sample.

The liver was investigated using multiphoton microscopy in the registration channel of NAD(P)H autofluorescence. For this purpose we used the LSM 880 laser scanning confocal microscope (Carl Zeiss, Germany) equipped with SPC 150 TCSPC FLIM module (Becker & Hickl GmbH, Germany), femtosecond Mai Tai HP laser (Spectra-Physics, USA), and oil immersion С-Apochromat W Korr objective lens (Carl Zeiss, Germany) with 40× magnification and 1.3 numerical aperture. The field of view size was 213×213 (1024×1024 pixels). A femtosecond Ti:Sa ultrashort-pulse laser provided excitation radiation with a repetition rate of 80 MHz and 140±20 fs pulse duration. NAD(P)H had a 750 nm excitation wavelength, signal registration in the range of 450–490 nm. Exciting radiation power was 6 mW.

### Statistical analysis

8–10 fields of view were obtained for each time point of the experiment on the model of liver slices and primary liver culture and FLIM parameters for 20–30 hepatocytes were determined for each field of view. The statistical analysis was conducted using R-Studio software. Statistical differences were assessed applying a pair-wise multiple comparison procedure. Differences in the mean values between the experimental groups were considered statistically significant at p≤0.05. Normality of distribution was determined using the Shapiro–Wilk test, equality of variances — the Fisher test, the paired t-test with the Bonferroni correction was applied for the pair-wise comparison.

## Results

### Assessment of the synthetized nanoparticle stability

**Figure 1.**
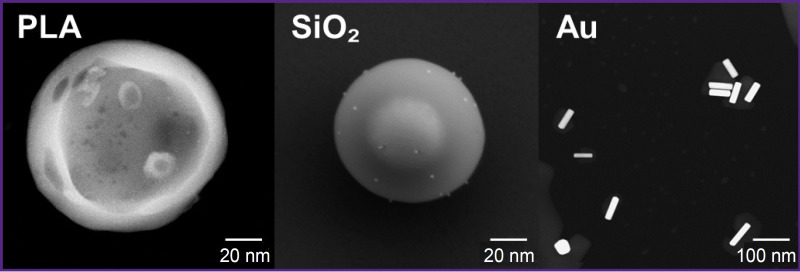
Microscopic visualization of the obtained nanoparticles Transmission electron microscopy of PLA NPs and Au NPs; scanning electron microscopy of SiO_2_ NPs

SiO_2_, PLA, and Au were selected as the materials for production of NPs. Microscopic images of all types of NPs are presented in Figure 1.

The main criterion, determining the size of the synthetized NPs, was their ability to pass through the liver vessel fenestrations and to remain in the cells for 24 h [16]. In this connection, the optimal NP size must be about 100 nm. The size of the synthetized NPs was 100±10 nm for SiO_2_, 100±5 nm for PLA, and 110±5 nm for Au.

### Assessment of nanoparticle distribution in the liver cells on the models of liver slices and primary liver cell culture

Based on the literature data and results of the preliminary series of the experiments we have chosen two concentrations of NPs, at which their accumulation in the cells caused minimal damage. Concentrations of 50 and 100 μg per 1 ml of cultural medium (μg/ml) were chosen for PLA NPs, for SiO_2_ NPs — 50 and 100 μg/ml, for Au NPs — 25 and 50 μg/ml (low Au NPs concentrations were chosen due to their identified toxicity). All further experimental series were carried out using these concentrations of NPs.

The model of liver slices allows taking into consideration the effect of interaction of various types of liver cells on biodistribution of NPs. The model of the primary liver cell culture makes it possible to determine more precisely the specific cell type, in which various types of NPs accumulate, and to assess the efficiency of their release from lysosomes.

For both models, all three types of Cy5-labeled NPs were visualized without any additional membrane staining.

**Figure 2.**
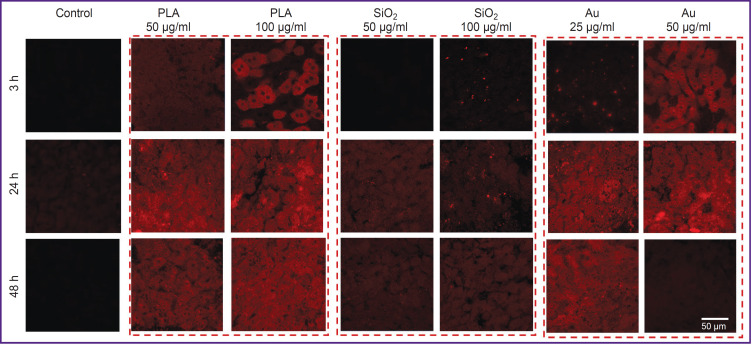
Biodistribution of Cy5-labeled nanoparticles in the liver slices Excitation of Cy5 at the 633 nm wavelength

It has been shown on the model of the liver slices that PLA NPs accumulated most effectively in the liver cells, mainly in hepatocytes. SiO_2_ NPs did not practically penetrate into the cells even when a high concentration was used (100 μg/ml), significant accumulation was observed only after 48 h. Au NPs also effectively accumulated in all types of the liver cells, however, a sharp reduction of Cy5 signal intensity was detected at the concentration of 50 μg/ml after 48 h of cultivation, which may be associated with toxic effect of NPs. The results of assessing the biodistribution of Cy5-labeled NPs are presented in Figure 2.

**Figure 3.**
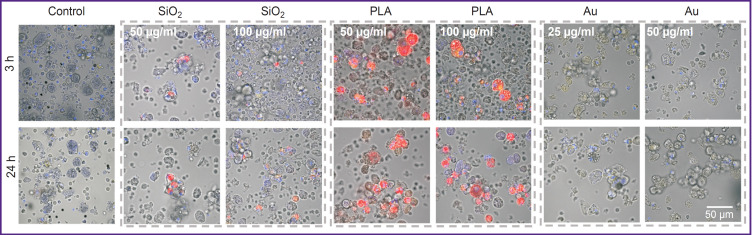
Biodistribution of Cy5-labeled nanoparticles in the primary culture of the liver cells Cy5-labeled nanoparticles (red): excitation — 750 nm, emission — 455–500 nm; LisoYellow lysotrecker, lysosome staining (yellow): excitation — 800 nm, emission — 371–421 nm; cell nuclei staining with Hoechst (blue): excitation — 405 nm, emission — 457 nm

The PLA NPs effectively accumulated in the cells, mainly in hepatocytes; their concentration was lower on macrophages. Hepatocytes are the most suitable localization for NP accumulation, since these particular cells make the greatest contribution to liver regeneration process. The SiO_2_ NPs accumulated insignificantly in the cells, mainly in macrophages, which is an unfavorable localization due to a high lytic activity of these cells. The Au NPs were hardly detected in the liver cells. Probably, they were washed out from the cells during sample preparation for imaging. It seems impossible to remove this preparation stage, which makes the model of primary liver cell culture unsuitable for the analysis of Au NP biodistribution. The results of NP biodistribution in the primary liver cell culture are presented in Figure 3.

### Metabolic imaging of liver slices and primary liver culture exposed to three types of nanoparticles

**Figure 4.**
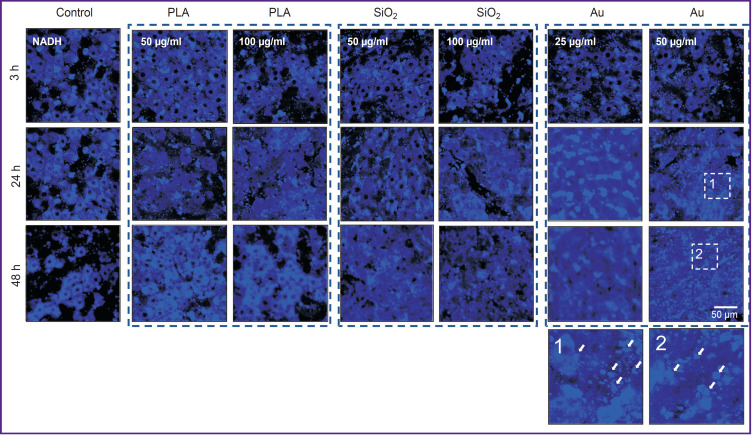
Autofluorescence intensity of the liver slices in the NADH channel affected by fluorescent label-free nanoparticles NADH autofluorescence excitation was at 750 nm wavelength. White arrows designate intracellular vesicles

When the liver slices were exposed to SiO_2_ (50 and 100 μg/ml) and PLA (50 and 100 μg/ml), the cell structure was not destroyed, and the values of NADH autofluorescence intensity did not actually differ from the control (without NP). After the action of Au at 25 and 50 μg/ml, apoptotic cells were detected (cells with multiple vesicles). The results of multiphoton microscopy of the liver slices are shown in Figure 4.

**Figure 5.**
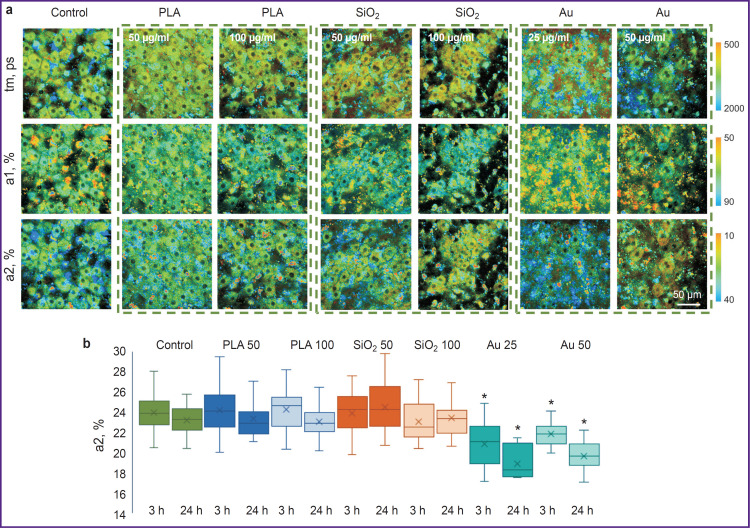
Metabolic imaging of liver slices exposed to the fluorescent label-free nanoparticles: (a) pseudostained FLIM images of liver slices after 3 h of cultivation; (b) histograms of distributing significance contributions of fluorescence lifetime for a bound form of NADH; * statistically significant difference from the appropriate time point in control (p≤0.05)

The FLIM parameters in the liver slices influenced by different types of NPs have been assessed. When exposed to PLA NPs, the values of all examined FLIM parameters (tm, a1, a2) did not actually differ from those in the control samples, which confirms the safety of this NP liver cell type (being especially important for hepatocytes). The SiO_2_ NPs also did not cause any significant changes in the FLIM parameters, however, in this case, this result was associated with ineffective accumulation of NPs in the cells. Finally, after the adding of Au NPs at two examined concentrations, we revealed heterogeneity of the liver tissue by the metabolic state of the cells, which shows the toxic effect of NPs on separate cells. For example, the value of a2 parameter decreased significantly after 24 and 48 h of cultivation with Au NPs (Figure 5 (b)), which designated a sharp reduction of oxidative phosphorylation intensity in the hepatic cells (the main metabolic pathway of hepatocytes). This hinders further application of Au NPs in the development of the strategy of stimulating liver regeneration. The results of FLIM analysis of the liver slices exposed to all types of NPs are presented in Figure 5.

**Figure 6.**
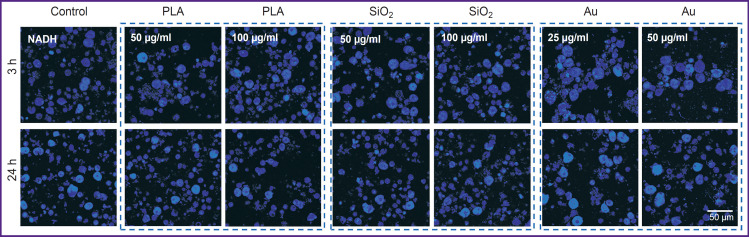
Intensity of autofluorescence of primary liver cell culture in the NADH autofluorescence channel exposed to the fluorescent label-free nanoparticles NADH autofluorescence excitation at the 750 nm wavelength

No changes occurred in the cell structure of the primary culture exposed to SiO_2_ (50 and 100 μg/ml) and PLA NPs (50 и 100 μg/ml), values of NADH autofluorescence intensity did not differ from the control (without NP). After adding of Au NPs, apoptotic cells were found at both concentrations (25 and 50 μg/ml). The results of multiphoton microscopy of the NADH of the primary cell culture exposed to all NP types are presented in Figure 6.

**Figure 7.**
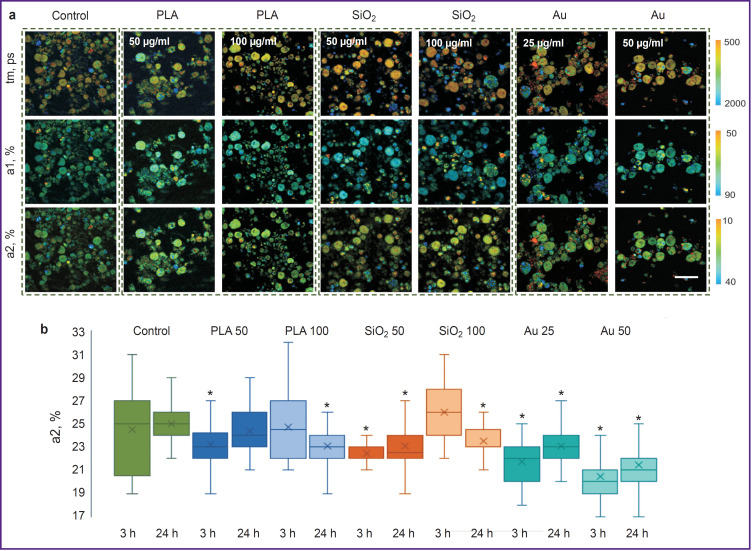
Metabolic imaging of the primary liver cell culture exposed to the fluorescent label-free nanoparticles: (a) pseudostained FLIM images of liver slices after 3 h of cultivation; (b) histograms of distributing significance contributions of fluorescence lifetime for a bound form of NADH; * statistically significant difference from the appropriate time point in control (p≤0.05)

Under the influence of PLA NPs, the values of all examined FLIM parameters (tm, a1, a2) in the primary liver cell culture differed insignificantly from those of control, however, there were detected single cells with the changed metabolic state (damaged hepatocytes). When exposed to SiO_2_ NPs at the concentration of 50 μg/ml, essential reduction of a2 (initial stages of hepatocytes damage) was noted. While at the concentration of 100 μg/ml, a sharp increase of a2 values was observed after 3 h of cultivation relative to the control, which may speak of the hepatocyte damage, as similar changes occur in toxic effect of paracetamol [17]. After 24 h of cultivation, a2 values decreased, necrotic hepatocytes appeared, confirming the toxic effect of these NPs. A significant decrease of a2 values at both concentrations occurred under the action of Au NPs, necrotic hepatocytes were present in the field of view (less than 20% of the total amount of cells in the field of view), which also pointed to the significant toxic effect of Au NPs. The results of the FLIM analysis of the primary live cell culture exposed to all types of NPs are presented in Figure 7.

In general, it can be concluded that only PLA NPs accumulate effectively in hepatocytes and do not change the metabolic state of hepatocytes.

### Assessment of cytotoxicity of three nanoparticle types on the models of liver slices and primary liver cell culture

**Figure 8.**
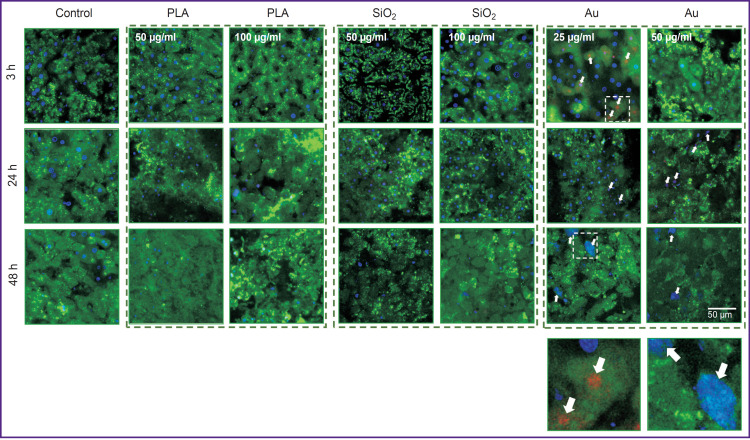
Viability of the cells in the liver slices exposed to nanoparticles Staining with calcein (green, live cells) and propidium iodide (red, dead cells); cell nuclei — Hoechst stain (blue)

The results of assessing the liver cell viability on the model of the liver slices are presented in Figure 8.

Polylactide and SiO_2_ did not cause any toxic effect on the liver slices. Up to 10–20% of dead cells appeared under the influence of Au NPs already after 3 h of culturing.

**Figure 9.**
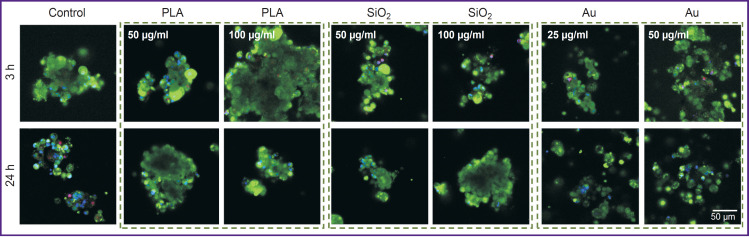
Viability of liver cells in the primary culture exposed to nanoparticles Staining with calcein (green, live cells) and propidium iodide (red, dead cells); cell nuclei — Hoechst stain (blue)

The results of cell viability assessment on the model of the primary culture are presented in Figure 9.

About 2% of dead cells were found in the primary culture of the liver cells even in the control samples after 3 h of culturing. The least amount of dead cells was observed under the adding of PLA NPs, whereas the greatest percentage was detected under the influence of Au PNs, especially at the concentration of 50 μg/ml.

## Discussion

Designing systems for stimulation of liver regeneration based on small bioactive molecules, especially microRNA, is an actively developing direction of scientific researches. One of the most promising carriers for these molecules is NPs. However, some problems regarding creation of such complexes remain unsolved. For example, a specific type of NPs capable of effective accumulation in the liver cells has not been identified. Exploration of the nanomaterial biodistribution pattern is of great importance for the assessment of possible application of these materials for biomedical purposes [1–7]. Besides, these biomaterials must be designed in such a way as to avoid long-term capturing by the cell lysosomes. A basic understanding of biodistribution of various NP types by the cells will make it possible to find the targets for bioactive molecules regulating different signaling pathways associated with liver regeneration [18, 19].

The liver acts as a system of biological filtration, which isolates 30–99% of the introduced NPs from the bloodstream, which makes the nanocontainers formation procedure much easier as it does not require additional modifications for targeted delivery.

In the present study, we have investigated three types of NPs, which seem to be the most perspective carriers for the small bioactive molecules: PLA, SiO_2_, Au [5].

Presently, SiO_2_ NPs are widely used for the delivery of diverse bioactive substantives. They possess a number of advantages such as biodegradation and biocompatibility. Besides, these NPs have universal physicochemical surface properties, while the synthesis procedure enables to easily control the size of the particles and their pores. Owing to this, the SiO_2_ NPs may be effectively loaded by the molecules of different types. Already now, siliсon NPs are applied to deliver chemical preparations for cancer therapy [20], optimization of delivery of immunostimulating molecules [21] and agents to enhance insulin permeability through the intestinal cells [22], and for the delivery of atrial natriuretic peptide to treat heart failure [23]. Moreover, it has been shown that even the introduction of “naked” SiO_2_ NPs increases protection of the liver against oxidative damage [24], contributes to fibrosis reduction [25]. However, large SiO_2_ NPs about 100–200 nm cause an inflammatory reaction in the mouse liver already 12 h after their administration into the systemic circulation [26]. The aim of our study was to assess specific features of NP biodistribution rather than their therapeutic properties. We have shown a low efficiency of SiO_2_ NP accumulation in the liver cells, and in this connection we believe that this type of particles is not suitable for creating complexes with bioactive molecules.

The Au NPs have been used for several decades for the delivery of various kinds of molecules [27, 28]. However, our study has demonstrated a marked toxic effect of Au NPs, which is in agreement with the investigations of other authors. The shape and size of Au NPs are known to influence their toxicity. Au NPs of 30 nm in size cause the greatest toxic effect, since they can penetrate the nucleus and damage DNA due to indirect oxidative stress. The main metabolic changes, leading to the apoptotic damage of the liver cells have been revealed [29], including triggering lipid peroxidation, rapid depletion of cytosol glutathione with concurrent increase of reactive oxygen species production, depolarization of transmembrane potential of mitochondria [30]. This strong effect of Au NPs on an oxidative state of hepatocytes is dose-dependent [31] making them inappropriate for creating complexes with bioactive molecules.

The PLA NPs represent a biocompatible and biodegradable copolymer, which presently is the most effective polymer carrier for delivering drugs and various molecules, and, most importantly, it has been approved for clinical application [32, 33]. In our work, we showed that PLA NPs accumulate maximally in hepatocytes already 24 h after their addition to the cultural media. Our results are in line with the data obtained by other authors. Thus, it has been established by Poilil Surendran et al. [33] on the models of mice with CCl_4_-induced fibrosis that PLA NPs accumulate in various liver cells and are suitable for passive and targeted drug delivery to treat liver fibrosis. El-Naggar et al. [34] have demonstrated the efficiency of targeted delivery of antioxidant molecules into the liver cells to reduce the oxidative stress in case of inflammation in diabetes type 1.

Hepatocytes are the most preferable type of cells for accumulation of NPs, since their division is the key factor in liver regeneration. Besides, absorption of NPs with a positive zeta potential by hepatocytes increases in contrast to macrophages, which absorb mainly negatively charged NPs [35]. This aspect was taken into consideration in our study: NPs were additionally covered with polyethylene glycol molecules thereby creating a weakly positive zeta potential for NPs [36].

## Conclusion

The biodistribution of three nanoparticles (PLA, SiO_2_, Au) in the liver cells has been investigated on the models of liver slices and primary liver cell culture. SiO_2_ nanoparticles are mainly accumulated by macrophages, which generate reactive oxygen species in a great amount, promoting destruction of bioactive molecules. Moreover, SiO_2_ nanoparticles impair the native metabolic state of hepatocytes. Au nanoparticles accumulate in all types of liver cells but possess a marked toxic effect, as indicated by the appearance of necrotic and apoptotic cells and a sharp change of the metabolic state of hepatocytes. Finally, PLA nanoparticles accumulate most effectively in the liver cells, mainly in hepatocytes, do not change their native metabolic state making this type of nanoparticles most promising for creating the systems for bioactive molecule delivery to stimulate liver regeneration.

## References

[1] Quek J., Chan K.E., Wong Z.Y., Tan C., Tan B., Lim W.H., Tan D.J.H., Tang A.S.P., Tay P., Xiao J., Yong J.N., Zeng R.W., Chew N.W.S., Nah B., Kulkarni A., Siddiqui M.S., Dan Y.Y., Wong V.W., Sanyal A.J., Noureddin M., Muthiah M., Ng C.H. (2023). Global prevalence of non-alcoholic fatty liver disease and non-alcoholic steatohepatitis in the overweight and obese population: a systematic review and meta-analysis.. Lancet Gastroenterol Hepatol.

[2] Yi P.S., Zhang M., Xu M.Q. (2016). Role of microRNA in liver regeneration.. Hepatobiliary Pancreat Dis Int.

[3] Zhao Z., Lin C.Y., Cheng K. (2019). siRNA- and miRNA-based therapeutics for liver fibrosis.. Transl Res.

[4] Wu P., Luo X., Wu H., Zhang Q., Dai Y., Sun M. (2020). Efficient and targeted chemo-gene delivery with self-assembled fluoro-nanoparticles for liver fibrosis therapy and recurrence.. Biomaterials.

[5] Lee S.W.L., Paoletti C., Campisi M., Osaki T., Adriani G., Kamm R.D., Mattu C., Chiono V. (2019). MicroRNA delivery through nanoparticles.. J Control Release.

[6] Taghizadeh S., Alimardani V., Roudbali P.L., Ghasemi Y., Kaviani E. (2019). Gold nanoparticles application in liver cancer.. Photodiagnosis Photodyn Ther.

[7] de Carvalho T.G., Garcia V.B., de Araújo A.A., da Silva Gasparotto L.H., Silva H., Guerra G.C.B., de Castro Miguel E., de Carvalho Leitão R.F., da Silva Costa D.V., Cruz L.J., Chan A.B., de Araújo Júnior R.F. (2018). Spherical neutral gold nanoparticles improve antiinflammatory response, oxidative stress and fibrosis in alcohol-methamphetamine-induced liver injury in rats.. Int J Pharm.

[8] Hyun J., Wang S., Kim J., Rao K.M., Park S.Y., Chung I., Ha C.S., Kim S.W., Yun Y.H., Jung Y. (2016). MicroRNA-378 limits activation of hepatic stellate cells and liver fibrosis by suppressing Gli3 expression.. Nat Commun.

[9] Kumar V., Mahato R.I. (2015). Delivery and targeting of miRNAs for treating liver fibrosis.. Pharm Res.

[10] Caldez M.J., Van Hul N., Koh H.W.L., Teo X.Q., Fan J.J., Tan P.Y., Dewhurst M.R., Too P.G., Talib S.Z.A., Chiang B.E., Stünkel W., Yu H., Lee P., Fuhrer T., Choi H., Björklund M., Kaldis P. (2018). Metabolic remodeling during liver regeneration.. Dev Cell.

[11] Stöber W., Fink A., Bohn E. (1968). Controlled growth of monodisperse silica spheres in the micron size range.. J Colloid Interface Sci.

[12] Chorny M., Fishbein I., Danenberg H.D., Golomb G. (2002). Lipophilic drug loaded nanospheres prepared by nanoprecipitation: effect of formulation variables on size, drug recovery and release kinetics.. J Control Release.

[13] Bartucci R., Åberg C., Melgert B.N., Boersma Y.L., Olinga P., Salvati A. (2020). Time-resolved quantification of nanoparticle uptake, distribution, and impact in precisioncut liver slices.. Small.

[14] Kegel V., Deharde D., Pfeiffer E., Zeilinger K., Seehofer D., Damm G. (2016). Protocol for isolation of primary human hepatocytes and corresponding major populations of nonparenchymal liver cells.. J Vis Exp.

[15] Zyuzin M.V., Antuganov D., Tarakanchikova Y.V., Karpov T.E., Mashel T.V., Gerasimova E.N., Peltek O.O., Alexandre N., Bruyere S., Kondratenko Y.A., Muslimov A.R., Timin A.S. (2020). Radiolabeling strategies of micron- and submicronsized core-shell carriers for in vivo studies.. ACS Appl Mater Interfaces.

[16] Zhang Y.N., Poon W., Tavares A.J., McGilvray I.D., Chan W.C.W. (2016). Nanoparticle-liver interactions: cellular uptake and hepatobiliary elimination.. J Control Release.

[17] Rodimova S., Mozherov A., Elagin V., Karabut M., Shchechkin I., Kozlov D., Krylov D., Gavrina A., Bobrov N., Zagainov V., Zagaynova E., Kuznetsova D. (2023). Label-free imaging techniques to evaluate metabolic changes caused by toxic liver injury in PCLS.. Int J Mol Sci.

[18] Chen X., Zhao Y., Wang F., Bei Y., Xiao J., Yang C. (2015). MicroRNAs in liver regeneration.. Cell Physiol Biochem.

[19] Forbes S.J., Newsome P.N. (2016). Liver regeneration — mechanisms and models to clinical application.. Nat Rev Gastroenterol Hepatol.

[20] Liu J., Chen Q., Feng L., Liu Z. (2018). Nanomedicine for tumor microenvironment modulation and cancer treatment enhancement.. Nano Today.

[21] Shahbazi M.A., Fernández T.D., Mäkilä E.M., Le Guével X., Mayorga C., Kaasalainen M.H., Salonen J.J., Hirvonen J.T., Santos H.A. (2014). Surface chemistry dependent immunostimulative potential of porous silicon nanoplatforms.. Biomaterials.

[22] Shrestha N., Araujo F., Shahbazi M.A., Mäkilä E., Gomes M.J., Herranz-Blanco B., Lindgren R., Granroth S., Kukk E., Salonen J., Hirvonen J., Santos H.A. (2016). Thiolation and cell-penetrating peptide surface functionalization of porous silicon nanoparticles for oral delivery of insulin.. Adv Funct Mater.

[23] Ferreira M.P., Ranjan S., Correia A.M., Mäkilä E.M., Kinnunen S.M., Zhang H., Shahbazi M.A., Almeida P.V., Salonen J.J., Ruskoaho H.J., Airaksinen A.J., Hirvonen J.T., Santos H.A. (2016). In vitro and in vivo assessment of heart-homing porous silicon nanoparticles.. Biomaterials.

[24] Liu Z., Li Y., Li W., Xiao C., Liu D., Dong C., Zhang M., Mäkilä E., Kemell M., Salonen J., Hirvonen J.T., Zhang H., Zhou D., Deng X., Santos H.A. (2018). Multifunctional nanohybrid based on porous silicon nanoparticles, gold nanoparticles, and acetalated dextran for liver regeneration and acute liver failure theranostics.. Adv Mater.

[25] Peng F., Tee J.K., Setyawati M.I., Ding X., Yeo H.L.A., Tan Y.L., Leong D.T., Ho H.K. (2018). Inorganic nanomaterials as highly efficient inhibitors of cellular hepatic fibrosis.. ACS Appl Mater Interfaces.

[26] Boey A., Ho H.K. (2020). All roads lead to the liver: metal nanoparticles and their implications for liver health.. Small.

[27] Siddique S., Chow J.C.L. (2020). Gold nanoparticles for drug delivery and cancer therapy.. Appl Sci.

[28] Amina S.J., Guo B. (2020). A review on the synthesis and functionalization of gold nanoparticles as a drug delivery vehicle.. Int J Nanomedicine.

[29] Khan H.A., Abdelhalim M.A., Alhomida A.S., Al-Ayed M.S. (2013). Effects of naked gold nanoparticles on proinflammatory cytokines mRNA expression in rat liver and kidney.. Biomed Res Int.

[30] Gao W., Xu K., Ji L., Tang B. (2011). Effect of gold nanoparticles on glutathione depletion-induced hydrogen peroxide generation and apoptosis in HL7702 cells.. Toxicol Lett.

[31] Sani A., Cao C., Cui D. (2021). Toxicity of gold nanoparticles (AuNPs): a review.. Biochem Biophys Rep.

[32] Devulapally R., Foygel K., Sekar T.V., Willmann J.K., Paulmurugan R. (2016). Gemcitabine and antisense-microRNA co-encapsulated PLGA–PEG polymer nanoparticles for hepatocellular carcinoma therapy.. ACS Appl Mater Interfaces.

[33] Poilil Surendran S., George Thomas R., Moon M.J., Jeong Y.Y. (2017). Nanoparticles for the treatment of liver fibrosis.. Int J Nanomedicine.

[34] El-Naggar M.E., Al-Joufi F., Anwar M., Attia M.F., El-Bana M.A. (2019). Curcumin-loaded PLA-PEG copolymer nanoparticles for treatment of liver inflammation in streptozotocininduced diabetic rats.. Colloids Surf B Biointerfaces.

[35] Wang H., Thorling C.A., Liang X., Bridle K.R., Grice J.E., Zhu Y., Crawford D.H.G., Xu Z.P., Liu X., Roberts M.S. (2015). Diagnostic imaging and therapeutic application of nanoparticles targeting the liver.. J Mater Chem B.

[36] Cornu R., Béduneau A., Martin H. (2020). Influence of nanoparticles on liver tissue and hepatic functions: a review.. Toxicology.

